# Metabolic Effects of Vitamin B1 Therapy under Overnutrition and Undernutrition Conditions in Sheep

**DOI:** 10.3390/nu13103463

**Published:** 2021-09-29

**Authors:** Mugagga Kalyesubula, Ramgopal Mopuri, Alexander Rosov, Guy Van Bommel, Hay Dvir

**Affiliations:** 1 Agricultural Research Organization—Volcani Institute, Institute of Animal Science, Rishon LeZion 7552809, Israel; mugagga.kalyesubula@mail.huji.ac.il (M.K.); mrg.bio2008@gmail.com (R.M.); rosov@volcani.agri.gov.il (A.R.); guyvb@volcani.agri.gov.il (G.V.B.); 2 Department of Animal Science, the Hebrew University of Jerusalem, Rehovot 7610001, Israel

**Keywords:** vitamin B1, obesity, micronutrient therapy, body weight, sheep

## Abstract

As a precursor for a universal metabolic coenzyme, vitamin B1, also known as thiamine, is a vital nutrient in all living organisms. We previously found that high-dose thiamine therapy prevents overnutrition-induced hepatic steatosis in sheep by enhancing oxidative catabolism. Based on this capacity, we hypothesized that thiamine might also reduce whole-body fat and weight. To test it, we investigated the effects of high-dose thiamine treatment in sheep under overnutrition and calorie-restricted undernutrition to respectively induce positive energy balance (PEB) and negative energy balance (NEB). Eighteen mature ewes were randomly assigned to three treatment groups (*n* = 6 each). The control group (CG) was administered daily with subcutaneous saline, whereas the T5 and T10 groups were administered daily with equivoque of saline containing 5 mg/kg and 10 mg/kg of thiamine, respectively. Bodyweight and blood biochemistry were measured twice a week for a period of 22 days under PEB and for a consecutive 30 days under NEB. Surprisingly, despite the strong effect of thiamine on liver fat, no effect on body weight or blood glucose was detectable. Thiamine did, however, increase plasma concentration of non-esterified fatty acids (NEFA) during NEB (575.5 ± 26.7, 657.6 ± 29.9 and 704.9 ± 26.1 µEqL^−1^ for CG, T5, and T10, respectively: *p* < 0.05), thereby favoring utilization of fatty acids versus carbohydrates as a source of energy. Thiamine increased serum creatinine concentrations (*p* < 0.05), which paralleled a trending increase in urea (*p* = 0.09). This may indicate an increase in muscle metabolism by thiamine. Reduction of fat content by thiamine appears more specific to the liver than to adipose tissue. Additional studies are needed to evaluate the potential implications of high-dose vitamin B1 therapy in muscle metabolism.

## 1. Introduction

Vitamin B1, also known as thiamine, is an essential precursor of thiamine pyrophosphate (TPP), a universal coenzyme for decarboxylation of α-keto acids with central roles in energy metabolism in all living organisms [1,2]. As a coenzyme in transketolase reactions, TPP also has a vital role in the pentose phosphate pathway, which is a significant route for the synthesis of nucleic acids, amino acids, steroids, lipids, neurotransmitters, glutathione and the supply of reduced NADP for various synthetic pathways [3].

Of particular importance to energy metabolism are the roles of TPP as a coenzyme for the mitochondrial α-ketoacid dehydrogenases: pyruvate dehydrogenase (PDH), branched-chain ketoacids dehydrogenase (BCKDH), and α-ketoglutarate dehydrogenase (α-KGDH). In the decarboxylation of pyruvate to acetyl-coenzyme A (acetyl-CoA) by PDH, the catalytic role of TPP couples glycolysis with terminal glucose oxidation in the tricarboxylic acid (TCA) cycle [4,5]. As a coenzyme for BCKDH, TPP has a vital role in the utilization of branched-chain amino acids, primarily from muscles, as a fuel source during starvation [6]. These roles emphasize the importance of thiamine bioavailability to partial and anaerobic catabolism of glucose and amino acids. The role of TPP in α-KGDH, which is a key enzyme for the turnover rate of the TCA cycle [7], emphasizes its significance to terminal oxidation of all three major macronutrients; carbohydrate, protein, and fat.

As a water-soluble vitamin, thiamine is readily excreted and exists in the body for a relatively short time. Therefore, maintaining proper physiology and metabolic viability requires a regular dietary intake of thiamine as a micronutrient, the insufficiency of which leads to common disorders of thiamine deficiency such as beriberi and Wernicke–Korsakoff syndrome [8,9,10].

Intriguingly, boosting thiamine physiological concentration has been shown to modulate metabolic physiology and pathophysiology. For instance, enhancing thiamine production in *Arabidopsis thaliana* increased carbohydrate oxidation in the TCA cycle [11]. In mammals, high-dose thiamine therapy countered circulating dyslipidemia in diabetic rats [12]; thiamine supplementation improved glucose tolerance in hyperglycemic individuals [13] and improved cardiovascular function [14], reduced serum pyruvate and lactate [15], and improved diabetic complications [16]. Recently, we have used a nutritional model of fatty liver in sheep [17] to show that high-dose vitamin B1 therapy prevents liver-fat accumulation driven by overnutrition [18]. Whether this substantial effect of thiamine on the liver fat content extends to extrahepatic tissues, with potential implications for weight control, is unclear. Herein, we tested the hypothesis that high-dose thiamine treatment would reduce body weight in sheep under both overnutrition and calorie-restricted undernutrition, i.e., during positive energy balance (PEB) and negative energy balance (NEB). To gain metabolic insight into the treatment effects, selected relevant circulating metabolites and minerals were monitored before and during the study.

## 2. Materials & Methods

### 2.1. Ethical Statement

All experimental procedures involving animals in this study were approved by the Volcani Institute Animal Care Committee (permit 843/19IL).

### 2.2. Animals and Experimental Design

**Figure 1:**
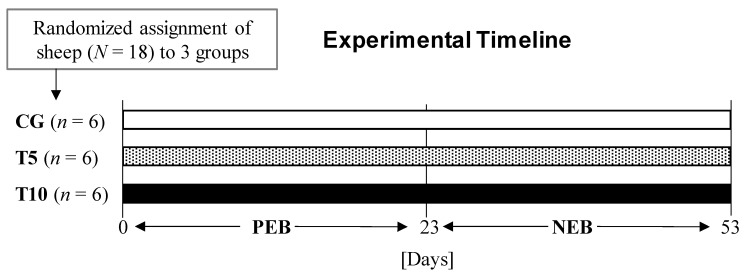
Experimental design and timeline. CG: Control group, T5 and T10: Thiamine treatment groups given a dose of 5 mg/kg and 10 mg/kg, respectively.

The animal experiments were carried out at the Volcani sheep experimental farm. A total of 18 ewes of the Affec–Assaf breed [19], with an average age of 2.7 ± 0.2 years and body weight of 77.7 ± 1.6 kg, were employed in a complete randomized trial consisting of three treatment groups (*n* = 6 each). The control group (CG) was treated with 2 mL of saline, while the thiamine treated groups T5 and T10 were treated respectively with 5 mg/kg and 10 mg/kg of thiamine dissolved in 2 mL of saline to evaluate dosage effect (Figure 1). To bypass potential ruminal metabolism of thiamine, the treatments were administered subcutaneously, five times a week [18]. The results of the thiamine treatment were evaluated in animals exposed to two nutritional states: PEB for the first 22 days and NEB for the consecutive 30 days (Figure 1). Animals were housed in individual cages for the entire experimental duration to control their individual feed intake. Under PEB, animals were fed a diet of concentrate and hay [17] that constituted 250% of their maintenance metabolizable energy requirements based on their body weights [20]. Under NEB, animals were given the same diet composition but at 50% of their metabolizable energy requirements. Feed intake was monitored daily, and body weights were measured twice a week.

### 2.3. Plasma and Serum Biochemical Analysis

Blood glucose and β-hydroxybutyrate (BHB) concentrations were measured using the FreeStyle Optium glucometer (Abbot Diabetes Care Ltd., Oxfordshire, UK) [21]. Plasma samples were isolated weekly from 5 mL of freshly drawn blood as described in [17] and stored at −20 °C until analysis of non-esterified fatty acids (NEFA) concentration using the Wako NEFA kit (Wako Chemicals, GmbH, Neuss, Germany).

Before the experiment and at the end of each nutritional phase, 5 mL of blood samples were collected into serum vacutainers (CAT serum Sep Clot Activator, VACUETTE, Greiner Bio-One, Kremsmünster, Austria) via venipuncture for serum biochemical analysis. Blood samples were left to clot at room temperature for ~3 h and then centrifuged at 1800× *g* for 15 min at 20 °C. Biochemical measurements of the ensuing sera were done using a Cobas Integra 400 Plus (Roche Diagnostics, Rotkruez, Switzerland).

### 2.4. Statistical Analysis

Data were analyzed by repeated-measures ANOVA with the linear mixed model approach in JMP (Version 15.1.0, SAS Institute Inc., Cary, NC, USA, 2019). For NEFA, glucose, weight, and weight gain, the model included: Treatment as a between-subject fixed factor with three levels (CG, T5, and T10), Nutritional State as a within-subject fixed factor, Treatment x Nutritional State, Time as a within-subject nominal fixed factor nested within Nutritional State, and Individual Animal as a random factor nested within Treatment. There were no effects for the baseline values, and therefore, they were excluded from the final statistical analysis. For data obtained from serum biochemical analysis, the model included: Treatment, Nutritional State, Treatment x Nutritional State, and Individual Animal as a random factor nested within Treatment. Post-hoc pairwise comparisons were carried out by the Tukey method for multiple hypotheses [22]. Unless otherwise indicated, data are presented as means ± SE. Statistical significance was accepted at *p* < 0.05, while statistical tendency was accepted at 0.05 *≤ p* ≤ 0.1.

Thiamine treatment was identical in both the PEB and NEB periods, so no carryover effects are expected. To assess potential nutritional carryover effects, the data were analyzed with and without exclusion of days 23–29, mimicking a nutritional washout period. These analyses yielded highly similar values and identical patterns of significant effects and trends. As no indication for carryover effects was observed, the data analyses presented herein correspond to all of the measured data points.

## 3. Results

### 3.1. Thiamine Increased Adipose Lipolysis during NEB but Did Not Affect Body Weight

The effect of high-dose thiamine treatment in mature ewes was investigated under two nutritional states, i.e., PEB and NEB using three treatment groups CG, T5, and T10 (Figure 1).

**Figure 2:**
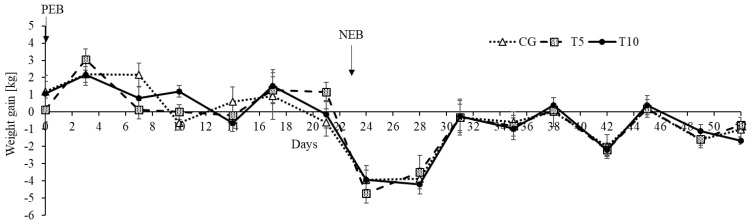
Weight gain response under positive (PEB) and negative (NEB) energy balance states in the control and thiamine treated groups. Effect of Treatment (*p* = 0.83), nutritional state (*p* < 0.0001), time (*p* < 0.0001), and Treatment by Nutritional State interaction (*p* = 0.94). CG: Control, T5: Thiamine treatment at 5 mg/kg, T10: Thiamine treatment at 10 mg/kg.

**Figure 3:**
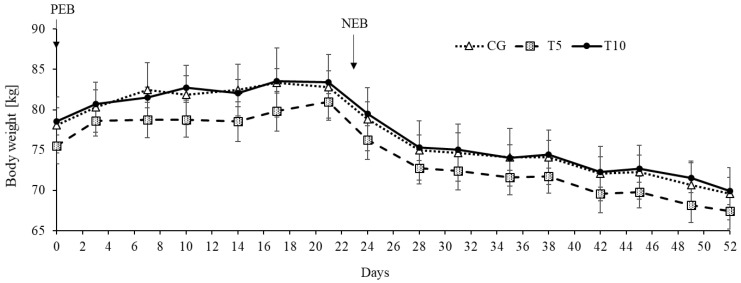
Weight response under positive (PEB) and negative (NEB) energy balance states in the control and thiamine treated groups. Effect of Treatment (*p* = 0.67), Nutritional State (*p* < 0.0001), time (*p* < 0.0001), and Treatment by Nutritional State interaction (*p* = 0.33). CG: Control, T5: Thiamine treatment at 5 mg/kg, T10: Thiamine treatment at 10 mg/kg.

As expected, the nutritional state affected sheep weight gain (*p* < 0.0001) since PEB led to weight gain while NEB led to weight loss (Figure 2). Likewise, the nutritional state affected the body weight (*p* < 0.0001), being lower during NEB compared to during PEB (Figure 3). There was no effect of thiamine on either the weight gain or the bodyweight (Figure 2 and Figure 3, Table 1). No effect was detected on weight gain normalized to the individual dietary intake of metabolizable energy (Table 1).

**Figure 4:**
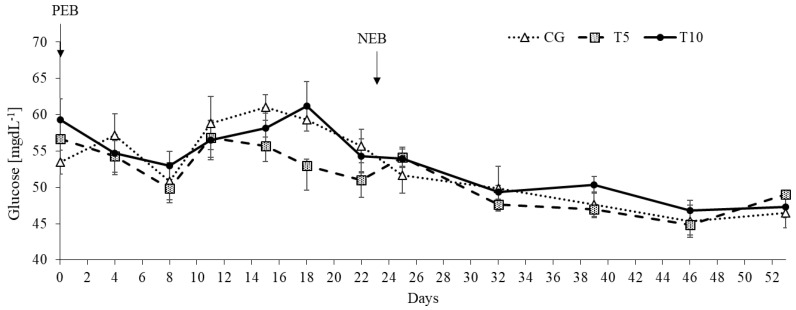
Blood glucose response under positive (PEB) and negative energy balance states (NEB) in the control and thiamine treated groups. Effect of Treatment (*p* = 0.54), Nutritional State (*p* < 0.0001), time (*p* < 0.0001), and Treatment by Nutritional State interaction (*p* = 0.06). CG: Control, T5: Thiamine treatment at 5 mg/kg, T10: Thiamine treatment at 10 mg/kg.

Similarly, the nutritional state affected blood glucose (*p* < 0.0001), being lower during NEB compared to PEB (Figure 4). There was a tendency for a Treatment by Nutritional State interaction (*p* = 0.06), likely because the variation in glucose values was higher during PEB than NEB (Figure 4). However, no significant overall effect of thiamine was detected on blood glucose.

**Figure 5:**
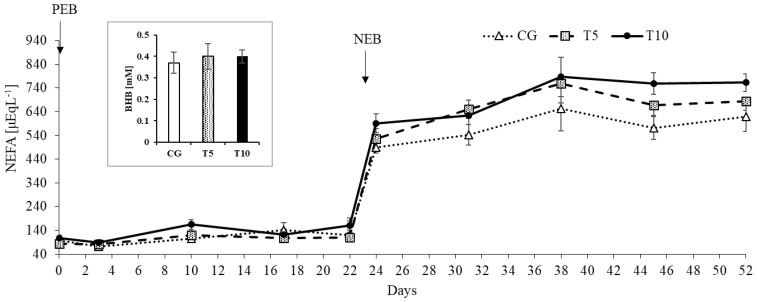
Non-esterified fatty acid (NEFA) response under positive (PEB) and negative (NEB) energy balance states in the control and thiamine treated groups. Effect of Treatment (*p* = 0.11), Nutritional State (*p* < 0.0001), Time (*p* < 0.0001), and Treatment by Nutritional State interaction (*p* < 0.01). CG: Control, T5: Thiamine treatment 5 mg/kg, T10: Thiamine treatment at 10 mg/kg. Inset is a graph of β-hydroxybutyrate (BHB) values at the end of NEB, showing no Treatment effect (*p* < 0.86).

As expected, the transition from PEB to NEB increased plasma NEFA concentrations (*p* < 0.0001; Figure 5). There was also a Treatment by Nutritional State interaction on the plasma NEFA concentrations (*p* = 0.01; Figure 5). This interaction reflects the increasing effect thiamine had on circulating NEFA during NEB (575.5 ± 26.7 µEqL^−1^ for CG 657.6 ± 29.9 for T5 and 704.9 ± 26.1 for T10; *p* < 0.05), which was not reflected during PEB (Table 1). Although thiamine increased NEFA concentrations during NEB, there were no Treatment effects on final blood BHB levels measured at the end of the NEB period, averaging at 0.37 ± 0.05, 0.4 ± 0.06, and 0.4 ± 0.03 mM for CG, T5, and T10, respectively (*p* = 0.86).

### 3.2. Thiamine Increased Serum Creatinine Concentrations

To get a broader perspective on the effect of the thiamine treatment, we measured several serum biochemical parameters under both PEB and NEB. Before the treatment initiation, all serum biochemistries were similar between the experimental groups (Table S1). Thiamine increased serum creatinine concentrations in a dose-dependent manner being highest in T10 (*p* < 0.05; Table 1). A similar trend of an increase in serum urea concentrations by thiamine was observed (*p* = 0.09). There was a Treatment by Nutritional State interaction for total serum bilirubin (*p* = 0.03), probably because total bilirubin concentrations were highest in CG during PEB, while during NEB, they were highest in T10 (Table 1). However, no apparent effect of thiamine on bilirubin was observed. The Nutritional State affected the concentration of several of the measured serum parameters. PEB increased the serum concentration of creatine kinase and urea (*p* < 0.05; Table 1); NEB increased the serum concentrations of albumin, total bilirubin, cholesterol, creatinine, and lactate dehydrogenase (*p* < 0.05; Table 1).

### 3.3. Thiamine Effects on Serum Mineral Concentrations

Before the experiment, all the measured serum parameters were similar between the experimental groups. Thiamine treatment increased serum calcium levels (*p* = 0.04; Table 1), but not in a dose-dependent manner, and the differences appear too small (<4% between T10 and CG) for physiological significance. Thiamine treatment also tended to affect serum phosphorus (*p* = 0.06; Table 1). The Nutritional State also affected some of the minerals; PEB increased Magnesium concentrations, whereas NEB increased the concentrations of Phosphorous, Sodium, and Chloride (*p* < 0.05; Table 1). However, these were unaffected by thiamine.

## 4. Discussion

Owing to the potential metabolic benefits of high-dose thiamine therapy, particularly with respect to reduction of liver steatosis [18] and blood glucose in diabetes [13], we investigated its effects on body weight, blood glucose, NEFA, and selected serum parameters under PEB and NEB. Although there was no effect of thiamine treatment on body weight, our results indicate that thiamine increased plasma NEFA concentrations during NEB. As a common surrogate for adipose lipolysis, the elevation of plasma NEFA indicates that thiamine modulated adipose metabolism to increase lipolysis. Although hepatic uptake of circulating NEFA is used to fuel ketogenesis, particularly in NEB [23,24,25], there were no treatment effects on final blood BHB concentrations. It is thus plausible that thiamine increased the hepatic oxidation capacity for complete NEFA oxidation through the TCA cycle, thereby countering the accumulation of acetyl-CoA as the precursor for ketone body synthesis [26]. It is also possible that part of the circulating NEFA was esterified in the liver as triglycerides and exported back to circulation in very-low-density lipoprotein (VLDL) particles, implying that the energy was more redistributed in the body than utilized. The latter possibility agrees better with the current observation of no effect for thiamine on whole-body weight, and with our previous indications for thiamine stimulation of liver gene expression favoring destabilization of lipid droplets and VLDL formation [18].

In the current study, no effect of thiamine on blood glucose levels was detected, although in our previous investigation, the glucose-lowering effect of thiamine treatment was significant [18]. Notably, the previous study investigated the impact of thiamine in hyperglycemic growing lambs, whereas the current study employed adult ewes of normal blood glucose levels. This may suggest that the effect of thiamine on blood glucose only becomes appreciable at hyperglycemic conditions, which is consistent with other studies in diabetic patients [13].

Thiamine treatment increased serum creatinine levels in a dose-dependent manner, which was paralleled by a trending rise in serum urea concentrations. Notably, the observed values remained within the normal range for creatinine (1.2–1.9 mg/dL) and urea (8–20 mg/dL) [27], indicating no potential renal dysfunction. Creatinine is a byproduct of muscle metabolism derived from creatine and creatine phosphate. The high energetic status of creatine phosphate plays an important role in energy metabolism in tissues of high and fluctuating energy demands, primarily skeletal and cardiac muscle [28]. Since creatinine formation is spontaneous and nonenzymatic, its levels are related to muscle mass. Therefore, serum creatinine is a common surrogate for muscle activity and mass [29,30,31,32]. The higher circulating creatinine in the thiamine-treated groups may potentially indicate an increased muscle mass or metabolic activity. Since the treatment duration in this study was relatively short, the rise in creatinine is more likely to reflect enhanced muscle creatine formation and utilization than a substantial increase in muscle mass.

The similar trending increase in urea concentrations by the thiamine treatment indicates an increased breakdown of amino acids. This is consistent with boosting muscle metabolism, potentially contributed by thiamine-mediated activity stimulation of BCKDH, the rate-limiting enzyme in branched-chain amino acid catabolism [33]. Studies in rats show that insufficient thiamine decreases the actual activity of BCKDH, thereby lowering BCAA oxidation [34]. Remarkably, high-dose thiamine therapy prevented hepatic steatosis induced by overnutrition in sheep [18], and restored reduced mitochondrial TPP-dependent enzymatic activities associated with traumatic brain injury even in the absence of thiamine deficiency [35], together suggesting that thiamine concentrations higher than the normal physiological levels may boost mitochondrial combustion, presumably though not necessarily exclusively by maximizing the oxidative capacity of TPP-dependent mitochondrial dehydrogenases.

Further body composition measurements and molecular studies are warranted to probe the specific modulation of muscle metabolism by high-dose vitamin B1 therapy.

## 5. Conclusions

In this study, the high-dose thiamine treatment modulated circulating NEFA concentrations, i.e., increased it during NEB, indicating enhanced adipose lipolysis. However, whole-body weight was unaffected. The increase of serum creatinine suggests that thiamine may have also increased muscle metabolism. Additional studies are required to assess the potential benefits of thiamine therapy for muscle activity and health.

## Figures and Tables

**Table 1:** Serum parameter, body weight, and non-esterified fatty acids (NEFA) analysis of the control (CG) and the thiamine treated sheep under positive and energy balance nutritional states.

Parameter	Positive Energy Balance				Negative Energy Balance				*p*-Values		
	CG	T5	T10	SEM	CG	T5	T10	SEM	TRT	NS	TRT × NS
Creatine Kinase, µL^−1^	181.3	184.7	185.0	10.3	124.3	195.7	137.2	17.2	0.43	0.04	0.14
Albumin, gdL^−1^	4.09	4.31	4.17	0.06	4.30	4.53	4.47	0.06	0.32	0.0002	0.73
AST, UL^−1^	93.5	126.7	113.6	9.1	90.6	214.7	221.6	38.9	0.23	0.11	0.47
Total bilirubin, mgdL^−1^	0.05	0.02	0.03	0.008	0.14	0.13	0.19	0.01	0.21	<0.0001	0.03
Calcium, mgdL^−1^	10.11	9.11	10.33	0.22	9.83	9.50	10.22	0.15	0.04	0.98	0.30
Cholesterol, mgdL^−1^	98.6	91.9	90.9	2.9	108.5	115.0	111.7	2.6	0.91	<0.0001	0.21
Creatinine, mgdL^−1^	0.72 *	0.83	0.88 *	0.03	0.82 ^§^	0.97	1.05 ^§^	0.04	0.05	0.0002	0.68
Urea, mgdL^−1^	39.2	45.7	46.1	1.7	23.8	26.8	27.6	0.94	0.09	<0.0001	0.66
GGT, µL^−1^	68.2	75.5	82.3	3.5	53.8	74.2	71.5	5.4	0.14	0.15	0.65
LDH, µL^−1^	798.8	1038.2	992.7	47.7	890.5	1624.3	1519.7	184.0	0.11	0.03	0.46
Magnesium, µL^−1^	2.17	2.41	1.72	0.09	1.75	1.72	2.10	0.05	0.62	<0.0001	0.21
Phosphorous, mgdL^−1^	4.38	6.17	4.86	0.37	6.11	7.72	5.58	0.40	0.06	0.001	0.44
Total Protein, gdL^−1^	7.58	7.42	7.51	0.13	7.37	7.44	7.55	0.1	0.93	0.56	0.45
Triglycerides, mgdL^−1^	9.42	9.53	10.57	1.29	7.17	9.33	12.21	0.93	0.36	0.86	0.56
Sodium, mmolL^−1^	144.3	145.2	145.0	0.4	149.3	148.8	148.8	0.44	0.98	<0.0001	0.64
Potassium, mmolL^−1^	5.04	5.13	5.23	0.09	5.19	5.29	5.26	0.09	0.72	0.34	0.90
Chloride, mmolL^−1^	101.4	101.9	101.5	0.42	109.0	106.7	107.8	0.7	0.68	<0.0001	0.37
NEFA, µEqL^−1^	109.8	104.7 ^§^	134.6 ^§^	5.3	575.5 *	657.6	704.9 *	16.8	0.11	<0.0001	0.01
Glucose, mgdL^−1^	57.1	53.4	56.3	0.54	48.2	48.5	49.6	0.64	0.54	<0.0001	0.06
Normalized weight gain, kgMCal^−1^	0.12	0.15	0.13	0.03	−1.20	−1.29	−1.25	0.13	0.93	<0.0001	0.93
Normalized body weight, kgMCal^−1^	13.0	13.0	13.1	0.03	61.7	61.5	62.4	0.17	0.67	<0.0001	0.02

* Means are different (*p* < 0.05) or ^§^ tend to be different (0.05 < *p* < 0.1), as analyzed within NS (Nutritional State) with Tukey correction. CG—Control. T5—Thiamine treatment at a dose of 5 mg/kg. T10—Thiamine treatment at a dose of 10 mg/kg. TRT—Treatment. AST: Aspartate aminotransferase. GGT: Gama-Glutamyltransferase. LDH: Lactate Dehydrogenase. NEFA: Non-esterified fatty acids. Normalized weight gain: Normalized weight gain was obtained by dividing the weight gain by the corresponding individual intake of metabolizable energy. Normalized body weight: Normalized body weight was obtained by dividing the weight by the corresponding individual intake of metabolizable energy.

## Data Availability

Not applicable.

## Supplementary Materials

The following are available online at https://www.mdpi.com/article/10.3390/nu13103463/s1, Table S1: Pretreatment values of serum parameters.

## References

[1] Voskoboyev A.I., Ostrovsky Y.M. (1982). Thiamin Pyrophosphokinase: Structure, Properties. And Role in Thiamin Metabolism. Ann. N. Y. Acad. Sci..

[2] Manzetti S., Zhang J., Van Der Spoel D. (2014). Thiamin function, metabolism, uptake, and transport. Biochemistry.

[3] Kerns J.C., Arundel C., Chawla L.S. (2015). Thiamin deficiency in people with obesity. Adv. Nutr..

[4] Ciszak E.M., Korotchkina L.G., Dominiak P.M., Sidhu S., Patel M.S. (2003). Structural basis for flip-flop action of thiamin pyrophosphate-dependent enzymes revealed by human pyruvate dehydrogenase. J. Biol. Chem..

[5] Hennig J., Kern G., Neef H., Spinka M., Bisswanger H., Hübner G. (1997). Molecular mechanism of regulation of the pyruvate dehydrogenase complex from E. coli. Biochemistry.

[6] Holecek M. (2001). Effect of starvation on branched-chain alpha-keto acid dehydrogenase activity in rat heart and skeletal muscle. Physiol. Res..

[7] Huang H.M., Zhang H., Xu H., Gibson G.E. (2003). Inhibition of the α-ketoglutarate dehydrogenase complex alters mitochondrial function and cellular calcium regulation. Biochim. Biophys. Acta-Mol. Basis Dis..

[8] Butterworth R.F. (1989). Effects of thiamine deficiency on brain metabolism: Implications for the pathogenesis of the wernicke-korsakoff syndrome. Alcohol Alcohol..

[9] Kril J.J. (1996). Neuropathology of thiamine deficiency disorders. Metab. Brain Dis..

[10] Brown G. (2014). Defects of thiamine transport and metabolism. J. Inherit. Metab. Dis..

[11] Bocobza S.E., Malitsky S., Araújo W.L., Nunes-Nesi A., Meir S., Shapira M., Fernie A.R., Aharoni A. (2013). Orchestration of Thiamin Biosynthesis and Central Metabolism by Combined Action of the Thiamin Pyrophosphate Riboswitch and the Circadian Clock in Arabidopsis. Plant Cell.

[12] Babaei-Jadidi R., Karachalias N., Kupich C., Ahmed N., Thornalley P.J. (2004). High-dose thiamine therapy counters dyslipidaemia in streptozotocin-induced diabetic rats. Diabetologia.

[13] Alaei Shahmiri F., Soares M.J., Zhao Y., Sherriff J. (2013). High-dose thiamine supplementation improves glucose tolerance in hyperglycemic individuals: A randomized, double-blind cross-over trial. Eur. J. Nutr..

[14] Shimon H., Almog S., Vered Z., Seligmann H., Shefi M., Peleg E., Rosenthal T., Motro M., Halkin H., Ezra D. (1995). Improved left ventricular function after thiamine supplementation in patients with congestive heart failure receiving long-term furosemide therapy. Am. J. Med..

[15] Falder S., Silla R., Phillips M., Rea S., Gurfinkel R., Baur E., Bartley A., Wood F.M., Fear M.W. (2010). Thiamine supplementation increases serum thiamine and reduces pyruvate and lactate levels in burn patients. Burns.

[16] Hammes H.P., Du X., Edelstein D., Taguchi T., Matsumura T., Ju Q., Lin J., Bierhaus A., Nawroth P., Hannak D. (2003). Benfotiamine blocks three major pathways of hyperglycemic damage and prevents experimental diabetic retinopathy. Nat. Med..

[17] Kalyesubula M., Mopuri R., Rosov A., Alon T., Edery N., Moallem U., Dvir H. (2020). Hyperglycemia-stimulating diet induces liver steatosis in sheep. Sci. Rep..

[18] Kalyesubula M., Mopuri R., Asiku J., Rosov A., Yosefi S., Edery N., Bocobza S., Moallem U., Dvir H. (2021). High-dose vitamin B1 therapy prevents the development of experimental fatty liver driven by overnutrition. Dis. Model. Mech..

[19] Gootwine E., Reicher S., Rozov A. (2008). Prolificacy and lamb survival at birth in Awassi and Assaf sheep carrying the FecB (Booroola) mutation. Anim. Reprod. Sci..

[20] National Research Council (2007). Nutrient Requirements of Small Ruminants: Sheep, Goats, Cervids, and New World Camelids.

[21] Kalyesubula M., Rosov A., Alon T., Moallem U., Dvir H. (2019). Intravenous Infusions of Glycerol Versus Propylene Glycol for the Regulation of Negative Energy Balance in Sheep: A Randomized Trial. Animals.

[22] Abdi H., Williams L.J., Salkind N.J. (2010). Tukey’s honestly significant difference (HSD) test. Encyclopedia of Research Design.

[23] McGarry J.D., Foster D.W. (1971). The regulation of ketogenesis from oleic acid and the influence of antiketogenic agents. J. Biol. Chem..

[24] McGarry J.D., Foster D.W. (1980). Regulation of hepatic fatty acid oxidation and ketone body production. Annu. Rev. Biochem..

[25] Adewuyi A.A., Gruys E., van Eerdenburg F.J. (2005). Non esterified fatty acids (NEFA) in dairy cattle. A review. Vet. Q..

[26] Bubber P., Ke Z.-J., Gibson G.E. (2004). Tricarboxylic acid cycle enzymes following thiamine deficiency. Neurochem. Int..

[27] Aiello S.E., Moses M.A., Allen D.G. (2016). The Merck Veterinary Manual.

[28] Brosnan J.T., Brosnan M.E. (2007). Creatine: Endogenous metabolite, dietary, and therapeutic supplement. Annu. Rev. Nutr..

[29] Andrews R., Greenhaff P., Curtis S., Perry A., Cowley A.J. (1998). The effect of dietary creatine supplementation on skeletal muscle metabolism in congestive heart failure. Eur. Heart J..

[30] Page A., Flower L., Prowle J., Puthucheary Z. (2021). Novel methods to identify and measure catabolism. Curr. Opin. Crit. Care.

[31] Patel S.S., Molnar M.Z., Tayek J.A., Ix J.H., Noori N., Benner D., Heymsfield S., Kopple J.D., Kovesdy C.P., Kalantar-Zadeh K. (2013). Serum creatinine as a marker of muscle mass in chronic kidney disease: Results of a cross-sectional study and review of literature. J. Cachexia. Sarcopenia Muscle.

[32] Istasse L., Van Eenaeme C., Gabriel A., Clinquart A., Maghuin-Rogister G., Bienfait J.M. (1990). The relationship between carcass characteristics, plasma hormones and metabolites in young fattening bulls. Vet. Res. Commun..

[33] Shimomura Y., Honda T., Shiraki M., Murakami T., Sato J., Kobayashi H., Mawatari K., Obayashi M., Harris R.A. (2006). Branched-chain amino acid catabolism in exercise and liver disease. J. Nutr..

[34] Navarro D., Zwingmann C., Butterworth R.F. (2008). Impaired oxidation of branched-chain amino acids in the medial thalamus of thiamine-deficient rats. Metab. Brain Dis..

[35] Mkrtchyan G.V., Üçal M., Müllebner A., Dumitrescu S., Kames M., Moldzio R., Molcanyi M., Schaefer S., Weidinger A., Schaefer U. (2018). Thiamine preserves mitochondrial function in a rat model of traumatic brain injury, preventing inactivation of the 2-oxoglutarate dehydrogenase complex. Biochim. Biophys. Acta-Bioenerg..

