# Supplementary material for: Metabolic Effects of Vitamin B1 Therapy under Overnutrition and Undernutrition Conditions in Sheep
Source: Nutrients. 2021 Sep 29;13(10):3463. doi: 10.3390/nu13103463 (PMC8540229; doi:10.3390/nu13103463)
Supplement: Supplementary file 1 [file nutrients-13-03463-s001.zip › nutrients-1356643-supplementary.pdf]

Supplementary Table S1: Pretreatment values of serum parameters

Parameter	CG[1]	T5[2]	T10[3]	SEM	Treatment (*P*-Values)
Creatine Kinase, μL^{-1}	180.2	151.5	154.8	7.8	0.28
Albumin, gdL^{-1}	4.23	4.34	4.15	0.07	0.51
Aspartate aminotransferase , UL^{-1}	96.1	89.6	97.1	3.7	0.69
Total bilirubin, mgdL^{-1}	0.04	0.03	0.05	0.008	0.55
Calcium, mgdL^{-1}	9.83	9.39	9.91	0.11	0.12
Cholesterol, mgdL^{-1}	93.2	92.0	91.7	3.4	0.98
Creatinine, mgdL^{-1}	0.81	0.85	0.93	0.03	0.17
Gama-Glutamyltransferase, μL^{-1}	60.3	65.3	67.5	1.5	0.14
Lactate Dehydrogenase, μL^{-1}	865.2	952	939.8	31.2	0.49
Magnesium, μL^{-1}	2.17	1.98	2.05	0.04	0.25
Phosphorous, mgdL^{-1}	5.13	5.86	5.01	0.31	0.50
Total Protein, gdL^{-1}	7.61	7.38	7.63	0.12	0.63
Triglycerides, mgdL^{-1}	11.2	10.4	16.4	1.7	0.38
Urea, mgdL^{-1}	33.9	37.3	35.5	1.0	0.38
Sodium, mmolL^{-1}	145.5	145.2	144.5	0.4	0.61
Potassium, mmolL^{-1}	5.23	5.18	4.97	0.06	0.19
Chloride, mmolL^{-1}	102.9	103.1	101.4	0.4	0.10

[1]CG - Control

[2]T5 – Thiamine treatment at a dose of 5mg/kg

[3]T10 – Thiamine treatment at a dose of 10mg/kg
